# Emodin for pulmonary fibrosis: a systematic review and meta-analysis of efficacy and molecular mechanisms

**DOI:** 10.3389/fmed.2025.1734512

**Published:** 2026-01-09

**Authors:** Xubang Zhou, Mingwei Sima, Zhengyuan Fang, Yan Cui, Moxuan Han, Yueqi Wang, Jiayu Liu, Yan Bi, Donghui Yue

**Affiliations:** 1 College of Basic Medicine, Changchun University of Chinese Medicine, Changchun, China; 2 College of Traditional Chinese Medicine, Chang Chun University of Chinese Medicine, Changchun, China; 3 College of Continuing Education, Changchun University of Chinese Medicine, Changchun, China

**Keywords:** emodin, pulmonary fibrosis, animal models, inflammation, oxidative stress, meta-analysis

## Abstract

**Objective:**

This systematic review and meta-analysis aimed to evaluate emodin’s therapeutic efficacy in animal models of pulmonary fibrosis (PF) and summarize its anti-fibrotic mechanisms, providing a theoretical basis for the application of emodin in the clinical treatment of fibrosis.

**Methods:**

A comprehensive literature search was conducted across 4 major international databases and 4 Chinese databases through July 2025. Study quality was assessed using the SYRCLE risk of bias tool. The mean difference (MD) or standardized mean difference (SMD) with 95% confidence intervals (CIs) was used to evaluate emodin’s effects on fibrosis severity, histopathological damage, inflammation, oxidative stress, and epithelial-mesenchymal transition (EMT).

**Results:**

Meta-analysis revealed emodin significantly attenuated PF across multiple scales [Szapiel score: SMD = −1.73, 95% CI: −2.02 to −1.43; Ashcroft score: SMD = −3.10, 95% CI: −4.40 to −1.79; fibrotic area: SMD = −4.97, 95% CI: −7.87 to −2.08]. Emodin substantially reduced hydroxyproline content (SMD = −1.91, 95% CI: −2.42 to −1.41) and collagen deposition, while improving alveolitis (SMD = −1.89, 95% CI: −2.21 to −1.57) and lung coefficients (SMD = −1.35, 95% CI: −2.06 to −0.65). Emodin also mitigated inflammation by reducing pulmonary levels of IL-6 (SMD = −3.86, 95% CI: −6.21 to −1.51), IL-1β (SMD = −3.21, 95% CI: −4.90 to −1.53), and TNF-*α* (SMD = −3.31, 95% CI: −3.96 to −2.67). Additionally, it attenuated oxidative stress and inhibited EMT by elevating SOD activity (SMD = 4.69, 95% CI: 3.59 to 5.80) while decreasing MDA (SMD = −3.58, 95% CI: −4.48 to −2.68) and TGF-β levels (SMD = −2.72, 95% CI: −3.41 to −2.02).

**Conclusion:**

Emodin effectively alleviates PF through reducing collagen deposition, attenuating inflammation, suppressing oxidative stress, and inhibiting EMT. Subgroup analyses indicated that heterogeneity across studies was partly attributable to variations in dosing regimens and animal species. Further investigation into the anti-fibrotic properties of emodin is warranted to facilitate its therapeutic development.

**Systematic review registration:**

CRD420251131483, https://www.crd.york.ac.uk/PROSPERO/view/CRD420251131483.

## 1 Introduction

Pulmonary fibrosis (PF) represents the end-stage manifestation of interstitial lung diseases triggered by diverse etiological factors, including infection, pharmacological agents, chemical exposures, and particulate matter (1, 2). Idiopathic pulmonary fibrosis (IPF), the most common form, has an incidence of 3–9 cases per 100,000 people per year, with higher rates in North America and Europe (3, 4). The disease has a poor prognosis. Median survival is 2–3 years without treatment and 3–4 years with current antifibrotic therapy, which is comparable to many malignancies (4, 5).

The pathogenesis of PF involves fibroblast proliferation, extracellular matrix (ECM) accumulation, and epithelial-mesenchymal transition (EMT). EMT is a process where epithelial cells lose their characteristics and acquire mesenchymal phenotypes, thereby contributing to fibroblast accumulation. Other key mechanisms include oxidative stress, inflammation, and ECM deposition (6–8). Although corticosteroids and immunosuppressive agents demonstrate therapeutic efficacy in early-stage PF, they fail to achieve complete disease reversal and are frequently associated with metabolism-related adverse effects (9–11). Current clinical management primarily relies on pirfenidone [which inhibits transforming growth factor-beta 1 (TGF-β) and TGF-β-induced collagen synthesis] and nintedanib (a tyrosine kinase inhibitor) for antifibrotic therapy; however, their clinical effectiveness remains suboptimal, and these agents are both costly and associated with considerable adverse effects (12–15). There is thus an urgent need for safer and more effective therapies.

Emodin (1,3,8-trihydroxy-6-methylanthraquinone) is an anthraquinone derivative isolated from *Rheum* species. It has diverse biological activities including antiviral (16), anti-inflammatory (17), anticancer (18), immunosuppressive (19), and pro-apoptotic properties (20). Furthermore, accumulating evidence highlights the therapeutic potential of emodin in preclinical fibrosis models across multiple organ systems, including the liver, pancreas, and lung (21–23). Multiple preclinical studies have investigated emodin in PF models, but several important gaps remain. First, the overall therapeutic efficacy across different experimental models and dosing regimens has not been quantitatively assessed. Second, findings on specific outcomes such as fibrosis scores and inflammatory markers appear inconsistent across studies. Third, the molecular mechanisms underlying its antifibrotic effects lack systematic integration. These limitations hinder the translation of preclinical findings to clinical applications.

Therefore, this study uses systematic review and meta-analysis to quantitatively evaluate the protective effects of emodin in experimental PF models and to systematically synthesize mechanistic pathways. This will provide preclinical evidence to inform future clinical investigation.

## 2 Methods

This systematic review was prospectively registered in the International Prospective Register of Systematic Reviews (PROSPERO) platform (registration number: CRD420251131483), and the protocol is accessible at https://www.crd.york.ac.uk/PROSPERO/view/CRD420251131483.

### 2.1 Search strategy

We conducted comprehensive searches of the following electronic bibliographic databases: PubMed, Web of Science, Embase, Cochrane Library, China National Knowledge Infrastructure (CNKI), Wanfang Database, VIP Database, and the Chinese Biomedical Database. The search encompassed animal experimental studies investigating emodin treatment for PF from January 2000 through July 2025. The search strategy incorporated the terms “pulmonary fibrosis,” “idiopathic fibrosis,” and “emodin,” combined using Boolean operators “AND” or “OR.” Literature collection was supplemented by manual screening of eligible citations from relevant articles to ensure comprehensive and accurate retrieval. Detailed search strategies are provided in Supplementary material. Reference lists of included studies were screened, and literature management was performed using Zotero software. Two independent reviewers screened eligible literature, eliminated duplicates and irrelevant articles, and extracted data. Discrepancies were resolved through consensus discussion.

### 2.2 Eligibility criteria

Eligibility criteria were formulated according to the PICOS framework: rats or mice with PF (Population, P); emodin (Intervention, I); animal models with PF receiving no treatment or placebo/vehicle control (Comparison, C); assessment of PF severity, alveolitis for pathological lung injury, collagen deposition, inflammatory response, oxidative stress levels, and EMT (Outcomes, O); preclinical controlled studies (Study design, S).

### 2.3 Inclusion criteria and outcome measures

Studies were included if they met the following criteria: (1) preclinical animal model studies (including rats and mice); (2) studies employing emodin as the intervention, without restrictions on route of administration, formulation, or treatment duration; (3) studies containing at least one PF control group, without restrictions on modeling method or modeling duration, with control animals receiving equal volumes of non-therapeutic vehicle or no intervention; (4) studies investigating PF as the target disease; (5) studies reporting at least one of the following outcome measures: pulmonary pathological injury indicators (assessment of PF severity and alveolitis), collagen deposition indicators (hydroxyproline content for quantitative meta-analysis; other collagen-related measures were analyzed qualitatively), EMT indicators (TGF-β content and expression levels), inflammation-related indicators [interleukin-6 (IL-6), interleukin-1β (IL-1β), and tumor necrosis factor-*α* (TNF-α) levels], and oxidative stress-related indicators [superoxide dismutase (SOD) and malondialdehyde (MDA) levels]. Subgroup analyses were performed for studies employing multiple dosages or different species. When measurement units were inconsistent across studies, appropriate conversions were performed for standardization.

### 2.4 Exclusion criteria

Studies were excluded if they met any of the following criteria: (1) duplicate data or studies unrelated to the research topic; (2) studies that did not report relevant results or failed to include specified outcome measures; (3) studies involving disease models combined with other pathological conditions.

### 2.5 Data extraction

During the data extraction phase, two reviewers independently screened the titles and abstracts of the identified studies to select those meeting the inclusion criteria and subsequently reviewed the full texts. Duplicate publications were identified by comparing author names, institutions, sample sizes, intervention details, and outcome data. When the same study was published in multiple languages (e.g., both Chinese and English versions), these were treated as a single study to avoid duplicate data inclusion. After discussion, duplicates or unrelated studies were excluded. The reviewers then extracted the data independently, compiling the information into an Excel data extraction table. This table included the following: (1) baseline information of the included studies (including first author and publication year); (2) species characteristics (including strain, gender, weight, and number of rats or mice); (3) drugs used to induce the PF model; (4) dosages and administration routes of emodin extract; (5) anesthesia methods; (6) outcome measures. In cases where units of the outcome measures were inconsistent across studies, a third reviewer was involved to standardize the units.

If experimental results were not reported in a study, the authors were contacted to retrieve the necessary data. When raw numerical data were unavailable from the text or tables, data were extracted from figures using GetData Graph Digitizer software (version 2.26), where data points were manually extracted by generating coordinates on the graph. To ensure reliability, data extraction from figures was performed independently by two reviewers, and any discrepancies were resolved through re-extraction and consensus discussion. Data presented as mean ± standard error of the mean (SEM) in the articles were converted to mean ± standard deviation (SD) for consistency. In studies with multiple intervention groups, the analysis focused solely on the PF control group and the emodin intervention group. Throughout these stages, we strictly adhered to the relevant guidelines in the Cochrane Handbook for Systematic Reviews of Interventions (Edition 6.5, 2024) (24).

### 2.6 Quality assessment and risk of bias

Two reviewers independently assessed the quality and risk of bias of the included studies using the SYRCLE animal risk of bias tool (25). The evaluation addressed six types of bias: selection bias, performance bias, detection bias, attrition bias, reporting bias, and other sources of bias, comprising a total of 10 questions. Each study was categorized into low risk, high risk, or unclear risk for each bias domain. Disagreements between reviewers were resolved through discussion, and if consensus could not be reached, a third reviewer was consulted. Inter-rater reliability was assessed using Cohen’s kappa statistic. “Other bias” was defined as any potential source of bias not covered by the other five domains, including industry funding, baseline imbalances, or inappropriate statistical methods. All included studies were assessed using Review Manager 5.4 software.

### 2.7 Statistical analysis

Meta-analysis results were visualized using forest plots generated by Review Manager 5.4 and Stata 17 software. The meta-analysis employed either a random-effects model (I^2^ > 50%) or a fixed-effects model (I^2^ < 50%) based on heterogeneity. Outcome measures were analyzed using continuous data. Effect sizes were calculated as standardized mean differences (SMD) using Hedges’ g (bias-corrected SMD) with 95% confidence intervals (95% CI), and heterogeneity was quantified using the Higgins index (I^2^) and chi-square statistics. A result with I^2^ > 50% or *p* < 0.01 was considered indicative of significant heterogeneity, and sensitivity analyses were performed by sequentially removing individual studies to assess the impact of heterogeneity. If a meta-analysis could not be performed, qualitative analysis was used to report the data. Additionally, subgroup analyses based on predefined criteria were conducted to evaluate the impact of different doses (≥40 mg/kg vs. <40 mg/kg) and species (rats vs. mice) on outcome heterogeneity. Sensitivity analyses for outcomes were performed using Stata 17 software, with publication bias assessed using funnel plots and Egger’s test, where *p* < 0.05 indicated the presence of bias. It should be noted that Egger’s test has limited reliability when fewer than 10 studies are included per outcome, and results should be interpreted with caution in such cases. The threshold for statistical significance was set at p < 0.05 for all outcomes, which aligns with conventional standards in meta-analysis. The heterogeneity threshold (*p* < 0.01) was maintained as a more conservative criterion for assessing between-study variation.

## 3 Results

### 3.1 Study selection

**Figure 1:**
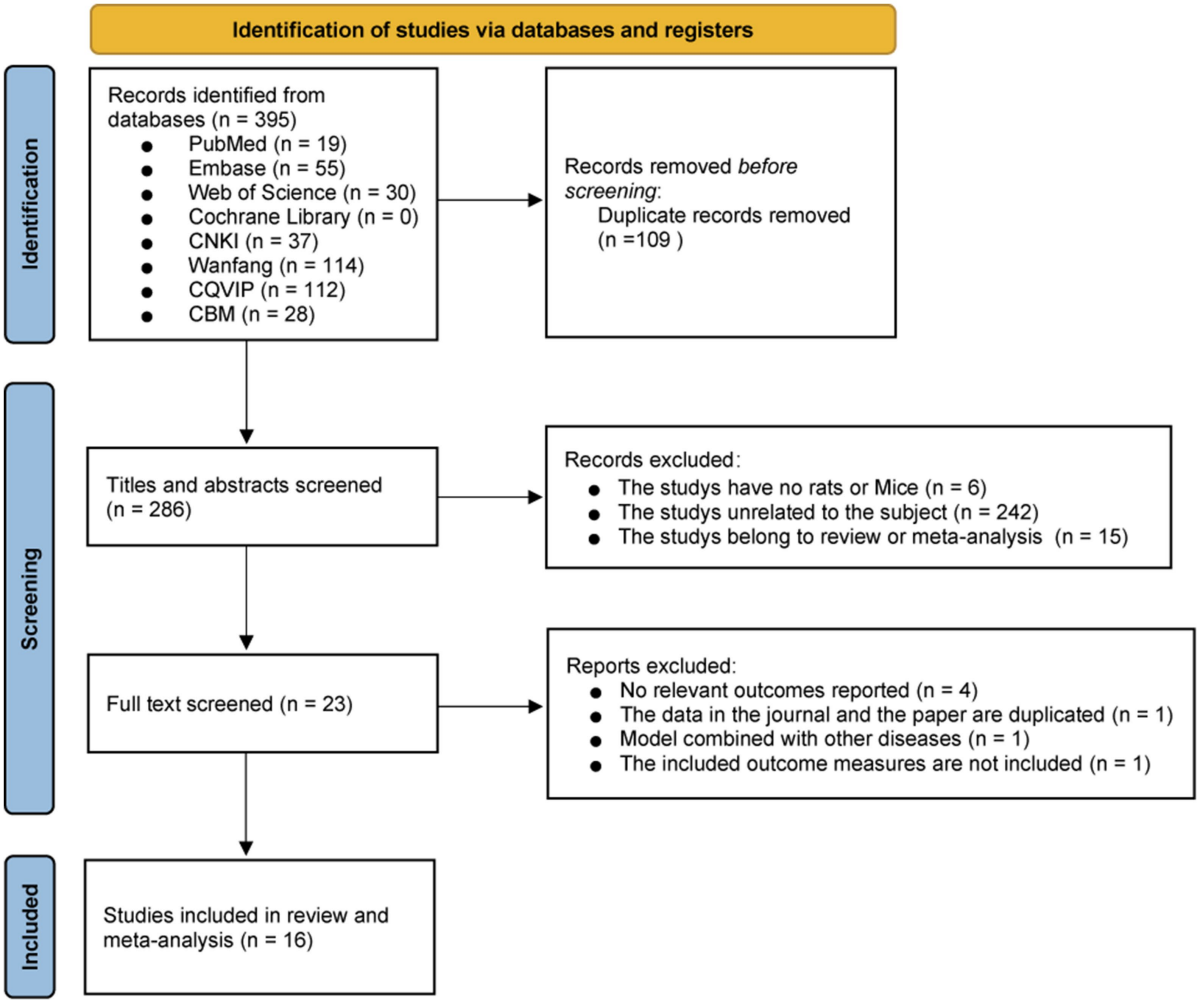
PRISMA 2020 flowchart for literature screening.

A total of 395 relevant studies were identified from eight databases, including 104 English-language studies and 291 Chinese-language studies. Specifically, PubMed contained 19 studies, Embase included 55, Web of Science had 30, Cochrane Library had no relevant studies, CNKI contained 37, Wanfang included 114, CQVIP contained 112, and CBM included 28. After excluding 109 duplicate studies, 286 studies remained. Further screening of titles and abstracts led to the exclusion of 6 studies that did not involve rats or mice, 242 studies irrelevant to the research topic, and 15 review or meta-analysis studies, leaving 23 studies for full-text review. Upon full-text assessment, 4 studies that did not report relevant outcomes, 1 study with duplicate data published in a different language, 1 study that combined other diseases in the model, and 1 study that did not include the specified outcome measures were excluded. Ultimately, 16 studies were included in the systematic review and meta-analysis (Figure 1).

### 3.2 Study characteristics

We included 16 studies involving 425 animals, of which two studies utilized both female and male rats (26, 27), while one study did not report mouse sex Chen et al. (21). Eleven studies used rats, comprising 228 Sprague–Dawley rats (≈ 54%) and 52 Wistar rats (≈ 12%), Sprague–Dawley rats were the most commonly used species. Five studies utilized C57Bl/6 mice (145 mice, 34%), with only one study using the C57Bl/6 J strain. The above statistics include the number of animals used across different dose groups. One study did not report mouse weight (28). The methods used to induce PF included intratracheal instillation of BLM in 10 studies, intratracheal instillation of silica suspension in 3 studies, intragastric administration of BLM in 1 study, intraperitoneal injection of LPS in 1 study, and paraquat solution administered by gavage in 1 study. The most common method for inducing PF was intratracheal instillation of BLM. The anesthesia methods varied widely, with 4 studies not specifying the details.

Among the 16 studies, emodin dosages ranged from 2 mg/kg to 80 mg/kg. Two studies used modified emodin nanoparticle formulations (Emo-NCs solution at 2 mg/kg and EDn solution at 5 mg/kg). Ten studies included 2–3 dose groups. The administration routes of emodin included oral gavage, intraperitoneal injection (28, 29), intratracheal aerosolization using a MicroSprayer® Aerosolizer (30), and intravenous injection (27). Details are provided in Table 1.

**Table 1:** Study characteristics of including studies (*n* = 16).

Study ID	Species, Sex	Experimental/Control (n)	Model	Anesthetic	Dosage of emodin (mg/kg)	Administration route	Outcomes
Guan et al. (34)	SD, male	6/6	BLM	Chloral hydrate	10, 20, 40	Oral gavage	I, IV, V, VII, IX
Liu et al. (45)	SD, male	10/10	BLM	Chloral hydrate	20, 80	Oral gavage	I, III, IV
Guan et al. (47)	SD, male	6/6	BLM	Chloral hydrate	20	Oral gavage	II, V
Tian et al. (37)	SD, male	5/5	BLM	NM	20	Oral gavage	I, II, IV, X, XI
Pang et al. (50)	C57BL/6, male	10/10	Silica	NM	25, 50	Oral gavage	I, III, IV, VII, IX
Yang et al. (42)	C57BL/6, male	8/8	BLM	Isoflurane	50	Oral gavage	I
Yang et al. (28)	C57BL/6, female	10/10	Silica	Sodium pentobarbital	20	Intraperitoneal injection	I, IV
Chen et al. (21)	C57Bl/6 J, NM	16/16	BLM	Sodium pentobarbital	5, 10, 20	Oral gavage	I, II, IV
Zhong et al. (46)	SD, male	10/10	BLM	Ether	20, 40, 80	Oral gavage	I, II, III
Liu et al. (38)	SD, male	10/10	BLM	Chloral hydrate	20, 80	Oral gavage	I, II, III, X, XI
Deng et al. (36)	SD, male	10/10	BLM	Chloral hydrate	20, 80	Oral gavage	I, III
Huang et al. (29)	SD, male	8/8	LPS	NM	12.5, 25, 50	Intraperitoneal injection	I
Li et al. (26)	Wistar rats, male/female	10/10	BLM	ether	20, 40, 80	Oral gavage	II, VI
Wang et al. (43)	SD, male	10/10	Paraquat	NM	40	Oral gavage	IV, VI, VII, IX
Zhang et al. (30)	C57Bl/6 J, male	5/5	BLM	Aivetin	Emo-NCs = 2; 20	Intratracheal aerosolization; oral gavage	I, II, VI
Sherekar et al. (27)	Wistar rats, male/female	6/6	Silica	Sodium thiopental	EDn = 5	Intravenous injection	I, II, III, IV, VI, VII, VII, IX, X, XI

BLM, bleomycin; Emo-NCs, emodin-loaded nanoparticles; EDn, emodin-loaded nanoparticles; PBS, phosphate buffered saline.

I: degree of fibrosis; II: Hydroxyproline (HYP); III: alveolitis; IV: TGF-β; V: pulmonary dynamic compliance; VI: lung coefficient; VII: IL-6; VII: IL-1β; IX: TNF-α; X: SOD; XI: MDA.

### 3.3 Quality assessment and risk of bias

**Figure 2:**
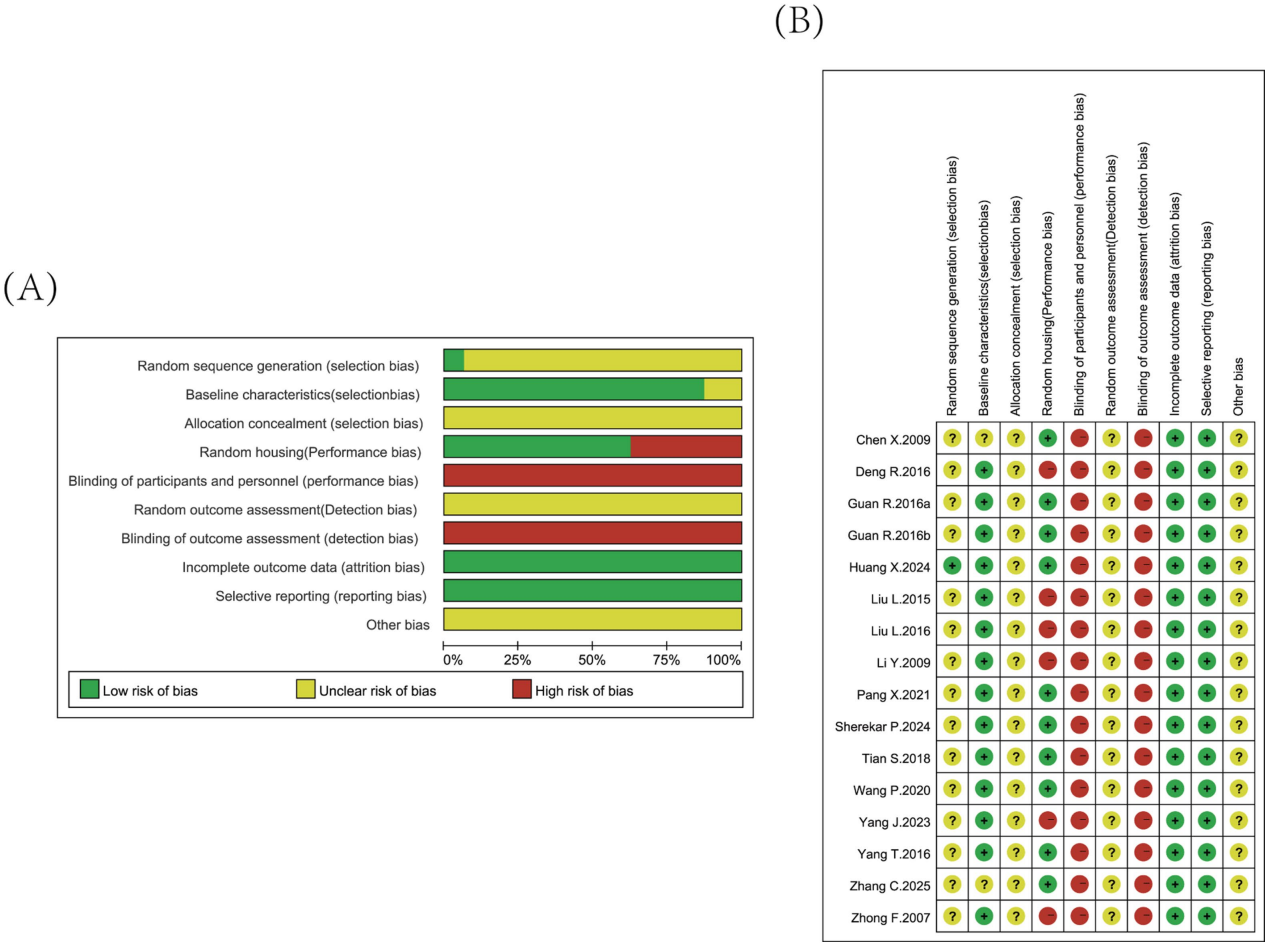
Using the SYRCLE risk of bias tool, bias risk assessment was conducted for the 16 included studies. Risk of bias graph **(A)**; risk of bias summary **(B)**.

Two reviewers independently assessed the risk of bias for each study using the SYRCLE risk of bias tool. One study (29) employed “randomization via random number tables” and was classified as low risk for sequence generation. However, while the remaining 15 studies mentioned randomization, the specific methods were not provided, resulting in an unclear risk rating. Two studies did not report initial body weight (30) and sex (21), leading to an unclear risk rating for baseline comparability. All 16 studies failed to provide sufficient information on whether appropriate allocation concealment procedures were implemented, resulting in an unclear risk rating for this domain. Ten studies employed random allocation of animals, while the remaining six did not specify this method and were rated as high risk. For blinding of the intervention implementer and random outcome assessment, all studies were evaluated as high risk and unclear risk, respectively. Additionally, the blinding of outcome assessors was considered a high risk due to unclear descriptions regarding the researchers’ knowledge of animal treatment. No studies reported incomplete outcome data or selective reporting, and as such, all were classified as low risk in these domains. For other sources of bias, all studies were rated as unclear risk. Notably, while some studies demonstrated low risk in certain areas, others showed unclear or even high risk in specific domains (Figure 2). Overall, despite some studies failing to report critical details such as animal age, sex, or allocation concealment methods, the majority provided comprehensive and satisfactory reporting in other areas.

### 3.4 Meta-analysis

#### 3.4.1 Degree of pulmonary fibrosis

Twelve studies assessed PF, with some studies including multiple dosage groups, and pathological injury was evaluated through three approaches. (1) Four studies involving 122 animals employed the blinded semi-quantitative scoring system described by Ashcroft (31); (2) six studies involving 268 animals used alternative scoring criteria (32); (3) two studies involving 20 animals evaluated fibrosis by calculating the percentage of fibrotic area.

**Figure 3:**
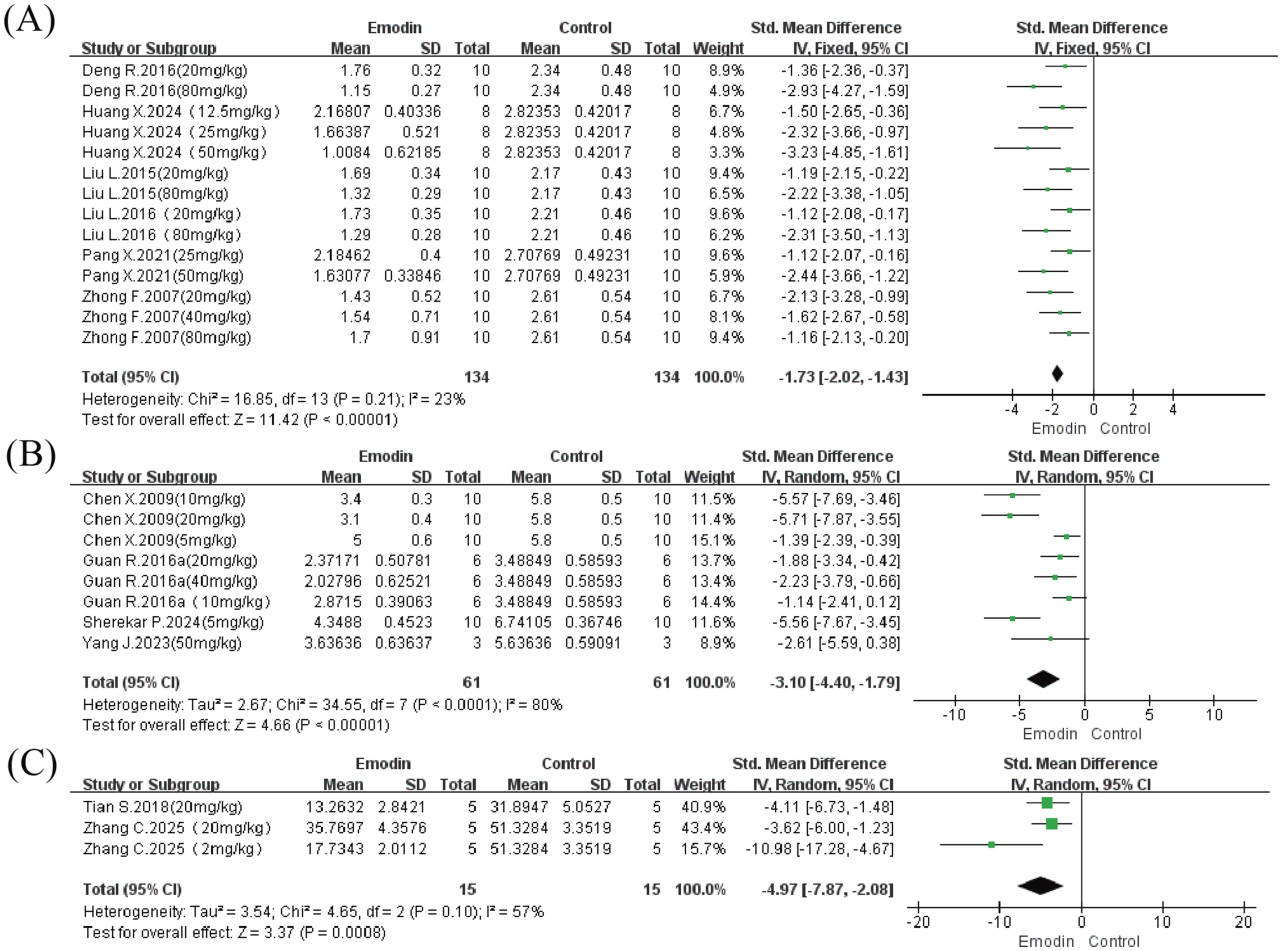
Forest map for assessing the degree of PF. Szapiel score **(A)**, from 0 to 3, from normal without lesion to severe lung tissue lesion; ashcroft score **(B)**, from 0 to 8, from normal without lesion to severe lung tissue lesion; percentage of PF area **(C)**.

Results indicated that (1) according to the Ashcroft scoring system, the degree of PF in the experimental group was significantly reduced compared to controls (SMD = −3.10, 95% CI: −4.40 to −1.79, *p* < 0.00001), although substantial heterogeneity was present (I^2^ = 80%); (2) for the Szapiel pathological scoring system, emodin similarly reduced the degree of PF (SMD = −1.73, 95% CI: −2.02 to −1.43, p < 0.00001) with lower heterogeneity (I^2^ = 23%); furthermore, assessment based on the percentage of fibrotic area also suggested that emodin confers beneficial effects on PF (SMD = −4.97, 95% CI: −7.87 to −2.08, *p* = 0.0008), though significant heterogeneity was observed due to the limited number of included studies (I^2^ = 57%) (Figure 3).

**Figure 4:**
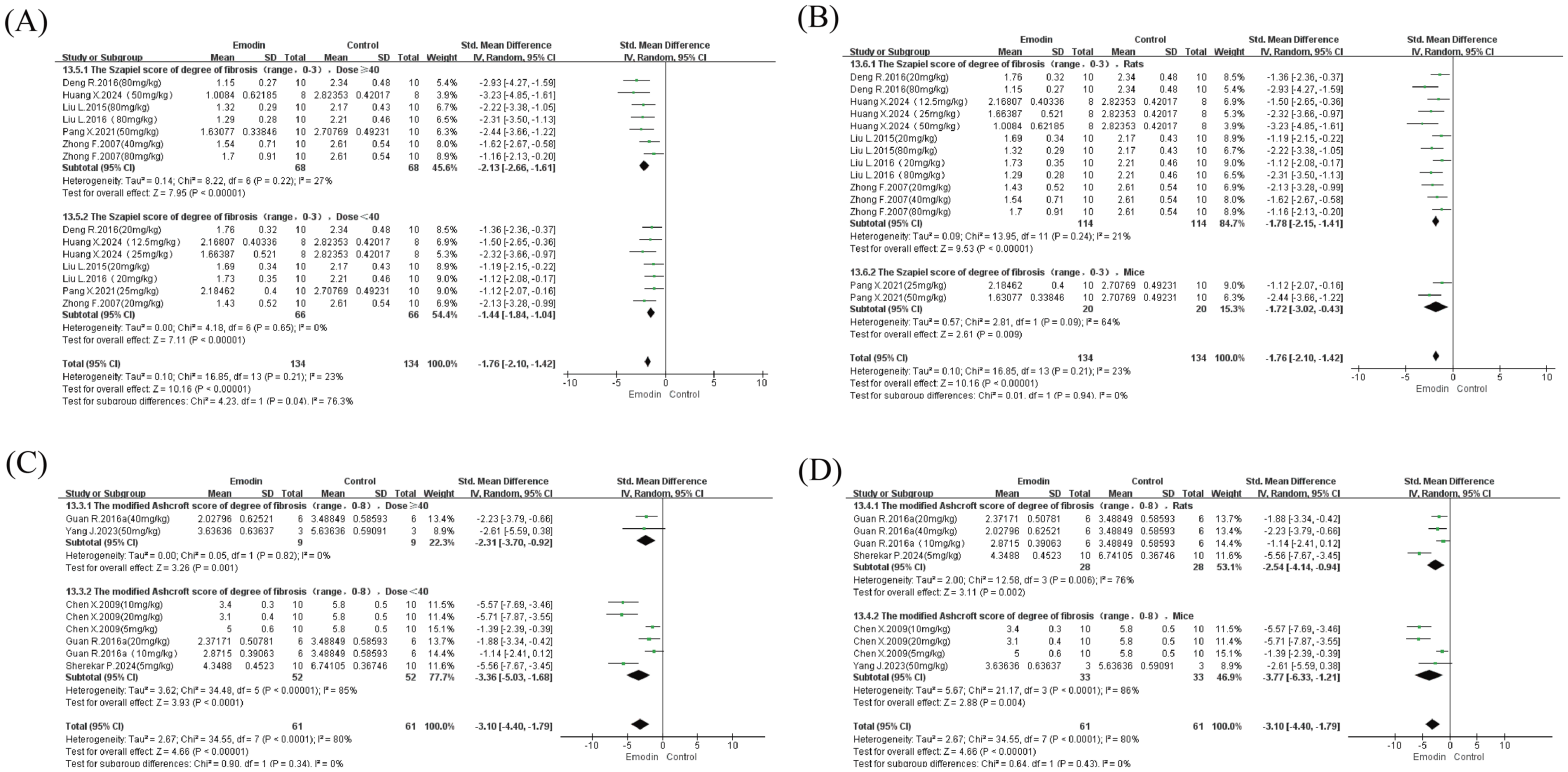
Subgroup analyses were conducted on Szapiel and Ashcroft scores related to the degree of PF, including different doses and species. Dose subgroup of Szapiel score **(A)**; species subgroup of Szapiel score **(B)**; dose subgroup of Ashcroft score **(C)**; species subgroup of Ashcroft score **(D)**.

We conducted subgroup analyses for studies employing two PF assessment methods (Szapiel and Ashcroft scoring systems), stratified by dosage (≥ 40 mg/kg and < 40 mg/kg) and species (rats and mice). Due to asymmetry in the number of studies across groups, results suggested that neither dosage nor species were primary sources of significant heterogeneity (Figure 4). Notably, however, heterogeneity was reduced in the Szapiel scoring system for emodin doses <40 mg/kg (I^2^ = 0%). In the species subgroup analysis, only one study using mice with two dosage groups was available (I^2^ = 64%), precluding meaningful comparison. Nevertheless, emodin doses ≥ 40 mg/kg and studies using rats appeared more beneficial for PF. For the Ashcroft scoring system, only two studies examined emodin doses <40 mg/kg (I^2^ = 0%), while subgroup analyses for doses ≥40 mg/kg and across species did not yield reduced heterogeneity. The limited number of studies employing the Ashcroft scoring method (only four studies with eight dosage groups) was insufficient to support robust subgroup analysis. Similarly, due to insufficient study numbers, analysis of fibrotic area percentage was not performed.

#### 3.4.2 Collagen deposition

**Figure 5:**
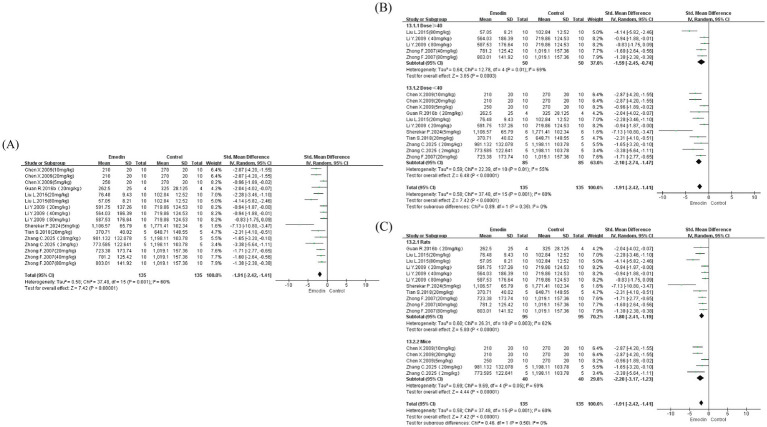
Forest map for evaluating HYP content. Forest map of HYP content **(A)**; dose subgroup of HYP **(B)**; species subgroup of HYP **(C)**.

Hydroxyproline (HYP) plays a pivotal role in collagen deposition, with excessive collagen accumulation being accompanied by elevated HYP levels, and inhibition of hydroxylase can mitigate fibrosis (33). Results from the eight included studies demonstrated that emodin significantly reduced HYP content in lung tissue (SMD = −1.91, 95% CI: −2.42 to −1.41, *p* < 0.00001), effectively alleviating collagen deposition, although considerable heterogeneity was observed (I^2^ = 60%) (Figure 5). Through sensitivity analysis, we excluded two studies employing different formulations and administration routes (27, 30), which resulted in reduced heterogeneity, suggesting that formulation and administration route contribute to heterogeneity. Review of the original studies did not reveal additional sources of heterogeneity.

Furthermore, subgroup analyses based on dosage and species indicated that emodin, whether administered at doses ≥ 40 mg/kg or < 40 mg/kg, and whether tested in rats or mice, attenuated PF progression (*p* < 0.01), though heterogeneity persisted within subgroups, precluding definitive identification of its sources. Similarly, sensitivity analysis excluding two low-dose studies with different administration routes and formulations (27, 30) reduced heterogeneity across subgroups (Supplementary Figure S1). However, given that only two such studies exist, further subgroup analysis could not be conducted.

#### 3.4.3 Alveolitis

**Figure 6:**
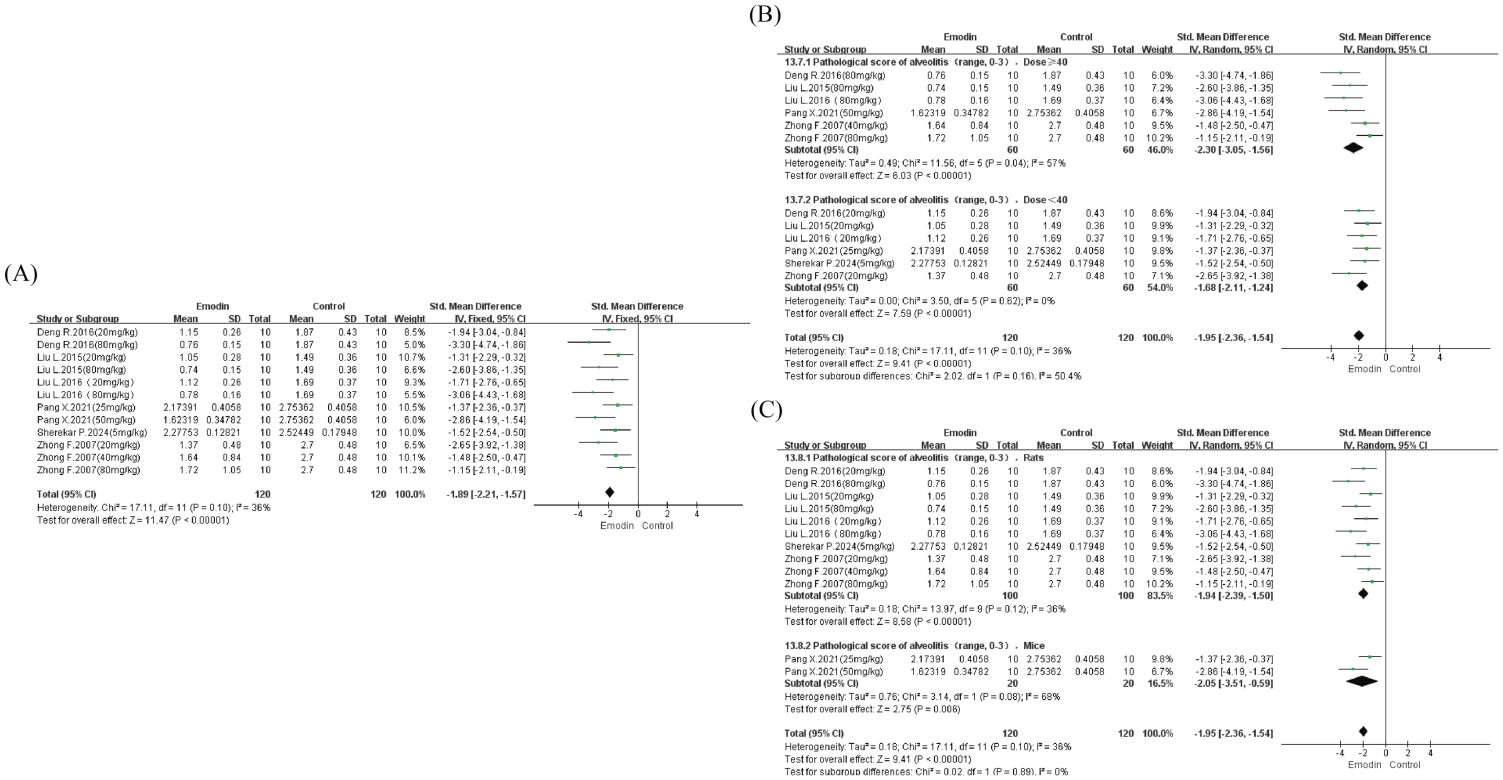
Forest map for evaluating inflammatory damage in lung tissue. Adopt Szapiel scoring systems. Forest map of alveolitis **(A)**; dose subgroup of alveolitis **(B)**; species subgroup of alveolitis **(C)**.

To evaluate the impact on inflammatory injury of lung tissue, we assessed six studies, which also included different dosages of emodin. Results demonstrated that emodin effectively ameliorated the occurrence of alveolitis and reduced pulmonary inflammatory injury (SMD = −1.89, 95% CI: −2.21 to −1.57, *p* < 0.00001). Although acceptable heterogeneity was present (I^2^ = 36%), we conducted subgroup analyses based on dosage and species to identify potential residual sources of heterogeneity. Results indicated that neither dosage nor species were major contributors to heterogeneity (overall I^2^ = 36%). Within the dosage subgroup, higher doses (≥ 40 mg/kg) appeared potentially more effective; however, only one study with two dosage groups examined mice, precluding accurate evaluation (Figure 6).

#### 3.4.4 TGF-β

**Figure 7:**
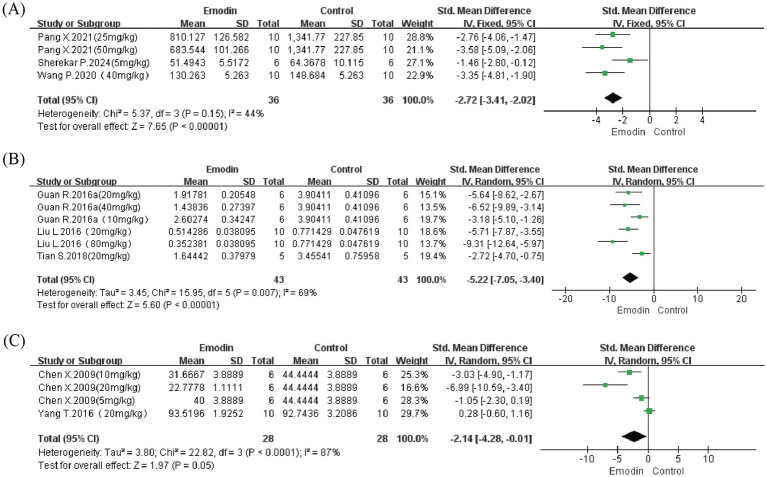
Forest map for evaluating the content and expression level of TGF-β in the lungs. Content of TGF-β in lung tissue **(A)**; level of TGF-β mRNA in lung tissue **(B)**; content of TGF-β in BALF **(C)**.

We further performed meta-analysis on TGF-β content and mRNA expression in lung tissue, as well as TGF-β levels in bronchoalveolar lavage fluid (BALF), to assess effects on PF and EMT. Results showed that while emodin demonstrated no significant effect on TGF-β levels in BALF (*p* = 0.05), it effectively reduced TGF-β content in lung tissue (SMD = −2.72, 95% CI: −3.41 to −2.02, *p* < 0.00001) and mRNA expression levels (SMD = −5.22, 95% CI: −7.05 to −3.40, *p* = 0.007) (Figure 7). However, due to the limited number of included studies (2–3 studies), further research incorporating additional studies is needed for more comprehensive evaluation.

#### 3.4.5 Pulmonary dynamic compliance

We analyzed respiratory function across three included studies. Results indicated that emodin did not consistently demonstrate the capacity to improve pulmonary dynamic compliance (SMD = 2.25, 95% CI: 0.00 to 4.49, *p* = 0.05), primarily attributable to one study (34) in which emodin at 10 mg/kg showed no significant difference compared to controls. Although respiratory function is not a primary outcome measure in PF, it warrants particular attention in advanced stages of the disease. Future studies are needed to evaluate the impact of PF on respiratory function, thereby providing indirect assessment of therapeutic efficacy (Supplementary Figure S2A).

#### 3.4.6 Lung coefficient

Relative changes in lung coefficient can provide indirect insight into pulmonary pathological alterations, and examining the lung coefficient (lung/body weight ratio) similarly offers indirect assessment of therapeutic efficacy. Analysis of four included studies revealed that emodin at various dosages effectively reduced lung coefficient (SMD = −1.35, 95% CI: −2.06 to −0.65, *p* = 0.0002). However, due to the limited number of included studies, considerable heterogeneity was observed in this outcome (I^2^ = 60%) (Supplementary Figure S2B).

#### 3.4.7 Inflammatory response

The progression of PF is inseparable from the involvement of inflammatory mediators in lung tissue; therefore, we analyzed the levels of IL-6 (2 studies), IL-1β (3 studies), and TNF-*α* (4 studies) in lung tissue. Our analysis demonstrated that emodin significantly reduced the levels of IL-6 (SMD = −3.86, 95% CI: −6.21 to −1.51, *p* = 0.001), IL-1β (SMD = −3.21, 95% CI: −4.90 to −1.53, p = 0.0002), and TNF-α (SMD = −3.31, 95% CI: −3.96 to −2.67, *p* < 0.00001) in lung tissue, thereby alleviating the inflammatory response (Supplementary Figures S2C–E). However, apart from the TNF-α outcome which exhibited low heterogeneity (I^2^ = 5%), the other two indicators displayed some heterogeneity due to insufficient numbers of includable studies.

#### 3.4.8 Oxidative stress

Oxidative stress accompanies all stages of PF progression. We analyzed oxidative stress outcome measures, including SOD and MDA; for other oxidative stress-related outcome indicators, this study lacked sufficient numbers for analysis. Results showed that in the analysis of three included studies, emodin increased SOD content in lung tissue (SMD = 4.69, 95% CI: 3.59 to 5.80, *p* < 0.00001) and decreased MDA content (SMD = −3.58, 95% CI: −4.48 to −2.68, p < 0.00001), with heterogeneity of I^2^ = 33% and I^2^ = 20%, respectively (Supplementary Figures S2F,G).

#### 3.4.9 Publication bias

**Figure 8:**
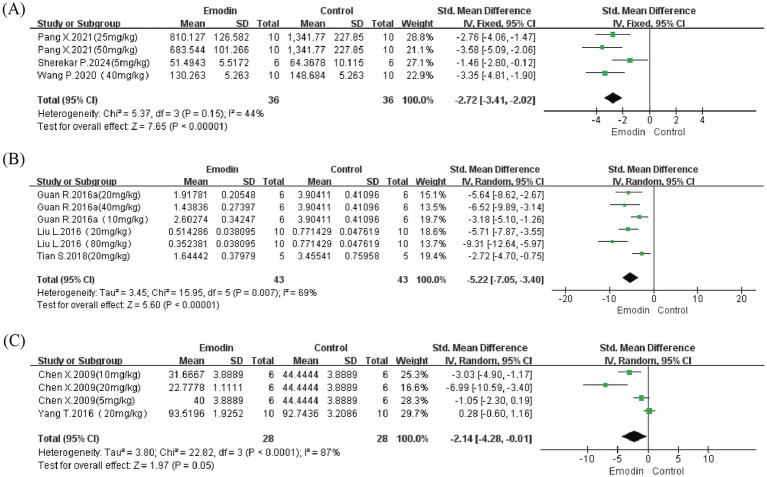
Publication bias represented by funnel plots. The Szapiel score for the degree of PF **(A)**; the Ashcroft score for the degree of PF **(B)**; HYP **(C)**; alveolitis **(D)**; content of TGF-β in lung tissue **(E)**; lung coefficient **(F)**.

We conducted further assessment of primary outcome measures, including the degree of PF (Szapiel and Ashcroft scores), HYP, alveolitis, TGF-β content in lung tissue, and lung coefficient. Funnel plots (Figure 8) were generated and Egger tests (Table 2) were performed to identify potential publication bias, excluding cases with possible visual discrepancies or those rendered meaningless by insufficient sample sizes. Statistical analysis revealed publication bias of statistical significance for all relevant outcomes (*p* < 0.05) except for TGF-β content in lung tissue (*p* = 0.316) and lung coefficient (*p* = 0.129). The literature included in this study comprised animal experiments, the majority of which reported positive results. Assessment of other related outcome indicators is detailed in Supplementary Figure S3 and Supplementary Table 1.

**Table 2:** Egger test.

Outcome	*t*	*p*	95% Conf. interval		No. of studies (containing different doses)
HYP	−5.39	0.000	−5.779526	−2.487119	16
Ashcroft score of degree of fibrosis (Ashcroft)	−3.00	0.024	−9.25226	−0.9430454	8
Ashcroft score of degree of fibrosis (Szapiel)	−9.33	0.000	−8.874059	−5.515094	14
Pathological score of alveolitis (Szapiel)	−45.83	0.000	−9.323618	−8.459018	12
Lung coefficient	−1.82	0.129	−10.01789	1.719206	7
Content of TGF-β in lung tissue	−1.33	0.316	−53.99775	28.56918	4

#### 3.4.10 Sensitivity analysis

**Figure 9:**
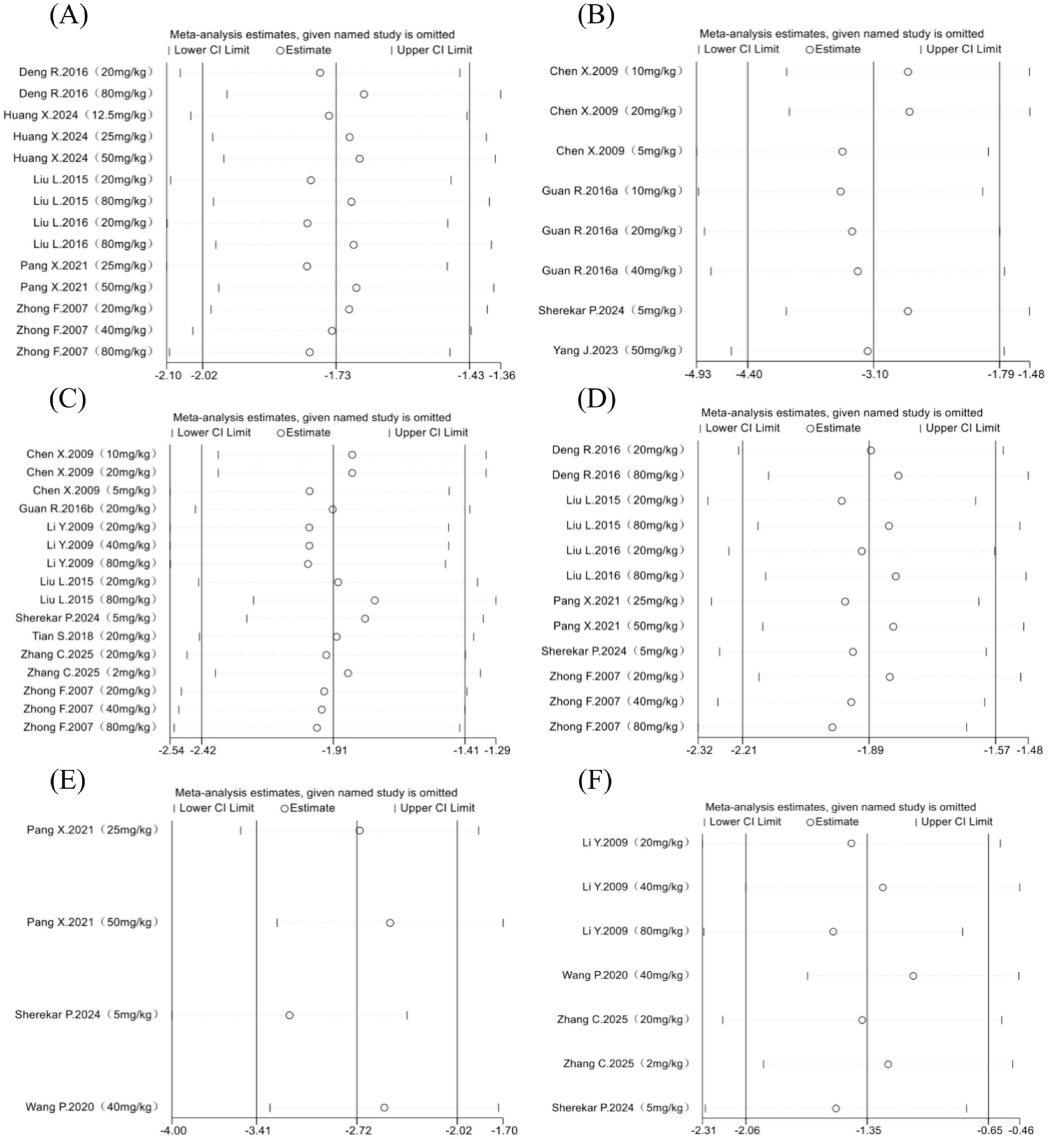
Outcome indicators sensitivity analysis. The Szapiel score for the degree of PF **(A)**; the Ashcroft score for the degree of PF **(B)**; HYP **(C)**; alveolitis **(D)**; Content of TGF-β in lung tissue **(E)**; lung coefficient **(F)**.

Sensitivity analysis was performed using Stata 17 software by sequentially excluding individual studies. The pooled SMD showed minimal variation across all outcomes (Szapiel score, Ashcroft score, HYP, alveolitis, TGF-β, and lung coefficient), with all iterations maintaining statistical significance (*p* < 0.001) and 95% confidence intervals consistently excluding zero. No single study disproportionately influenced the pooled estimates, confirming the stability and reliability of the findings (Figure 9). Other outcome indicators are detailed in Supplementary Figure S4.

## 4 Discussion

### 4.1 Summary of evidence

Prior to initiating this study, we found no comprehensive evaluation of emodin in treating PF; therefore, we undertook this work. We included 16 studies using preclinical animal models, involving 425 animals with PF. Of these, 15 studies were conducted in China between 2007 and 2025, and one study was completed in India in 2024. The overall reporting quality demonstrated comprehensiveness and standardization, with methodological assessment revealing potential risks of bias. Our findings indicate that emodin treatment ameliorated pulmonary histopathological damage related to the degree of PF and alveolitis. Additionally, emodin alleviated collagen deposition by reducing HYP content in lung tissue and decreased both TGF-β content and mRNA expression levels in lung tissue, reflecting emodin’s capacity to block the PF process and inhibit EMT. Regarding anti-inflammatory effects, emodin application significantly reduced levels of relevant inflammatory factors (IL-6, IL-1β, and TNF-*α*), effectively mitigating the inflammatory response. Emodin also demonstrated notable potential in antioxidant activity, as evidenced by elevated SOD and reduced MDA in lung tissue. While our meta-analysis focused on these measurable endpoints, individual studies within our review also explored underlying molecular mechanisms.

### 4.2 Potential mechanisms of emodin in treating pulmonary fibrosis

**Figure 10:**
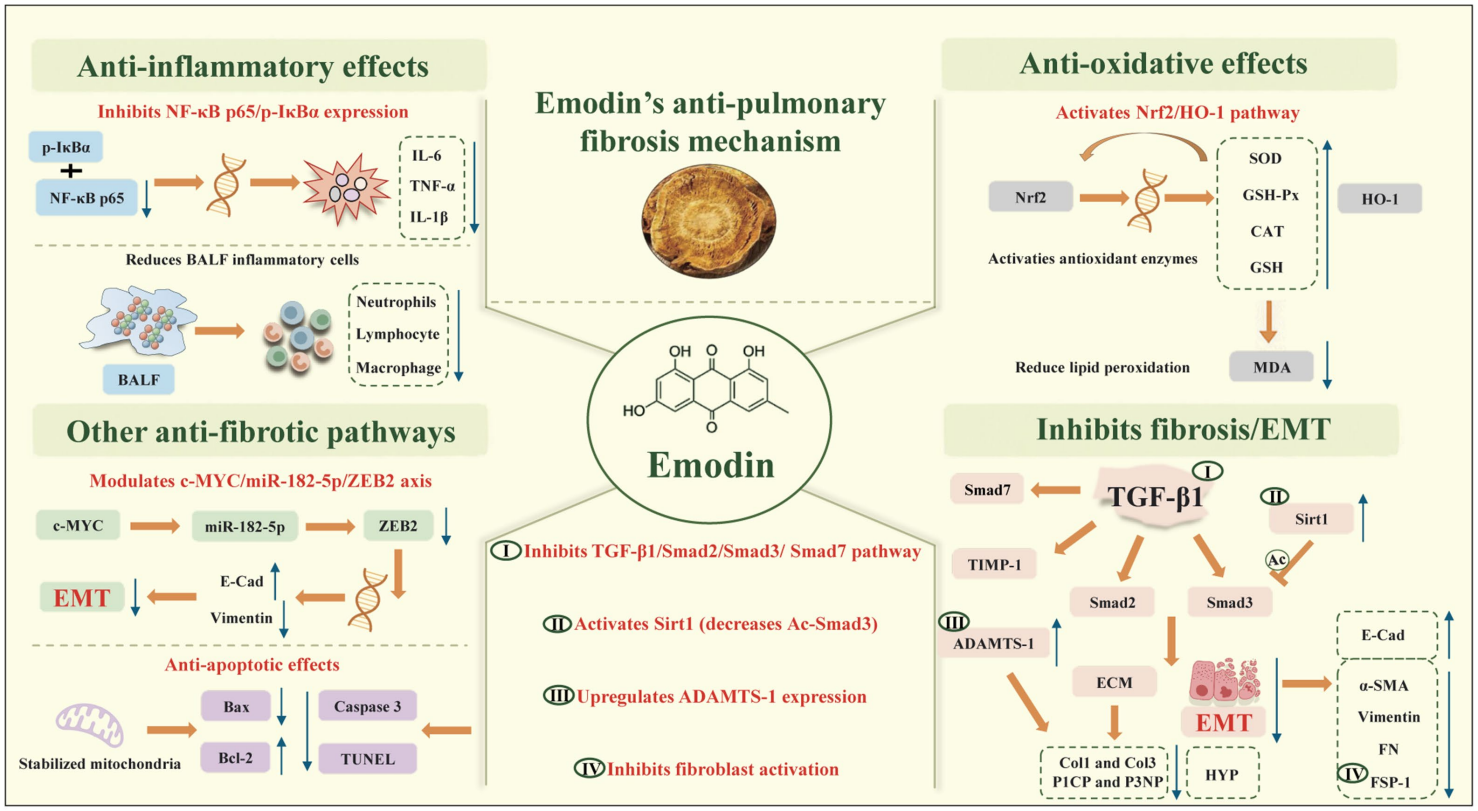
Potential mechanisms underlying emodin treatment of PF. Emodin exerts protective effects through four major pathways: **(A)** anti-inflammatory action via NF-κB inhibition and reduced cytokine production (IL-6, TNF-*α*, IL-1β); **(B)** anti-oxidative effects through Nrf2/HO-1 activation and enhanced antioxidant enzyme activities (SOD, GSH-Px, CAT, GSH); **(C)** inhibition of fibrosis/EMT by suppressing TGF-β1/Smad signaling, reducing fibroblast activation and ECM deposition while maintaining E-cadherin expression; and **(D)** additional anti-fibrotic effects via Sirt1/Ac-Smad3, ADAMTS-1 upregulation, c-MYC/miR-182-5p/ZEB2 axis modulation, and mitochondrial stabilization with anti-apoptotic activity.

Our evaluation of this study confirmed our findings that emodin exerts therapeutic effects on PF through modulation of diverse mechanisms, including anti-inflammatory, antioxidant, anti-apoptotic pathways, and inhibition of EMT (Figure 10). Beyond the outcomes directly synthesized in our meta-analysis, individual studies within our review investigated specific molecular mechanisms that may contribute to emodin’s anti-fibrotic effects. These findings were not quantitatively meta-analyzed due to limited replication across studies or heterogeneity in measurement methods, and therefore represent hypotheses requiring validation through future research rather than established conclusions from our systematic review.

Persistent inflammation and oxidative stress constitute the fundamental driving forces underlying the onset and progression of PF (35). The inflammatory cascade is initiated by transcription factors such as NF-κB, which rapidly respond and induce massive release of inflammatory mediators. Emodin significantly reduced the levels and mRNA expression of pro-inflammatory cytokines (including IL-6, TNF-*α*, IL-1β, and IL-4) in BALF and lung tissue (27, 34, 36). Mechanistically, emodin suppressed NF-κB p65 expression and IκBα phosphorylation, thereby blocking nuclear translocation of inflammatory signals (37). Individual studies within our analysis attributed this to inhibition of NF-κB p65/p-IκBα signaling, though this mechanism itself was not quantitatively synthesized. The consistent reduction in inflammatory cytokines across studies provides robust evidence for emodin’s anti-inflammatory capacity. Furthermore, as a key transcription factor in antioxidant defense, emodin activated the Nrf2 signaling pathway to promote nuclear translocation and induced expression of downstream antioxidant and detoxifying enzymes, including HO-1, SOD, GSH, GSH-Px, and CAT (37, 38). Enhanced activity of these enzymes effectively scavenged reactive oxygen species (ROS), thereby substantially decreasing MDA content, a lipid peroxidation product, and reducing tissue damage (27). Notably, negative crosstalk exists between Nrf2 and the NF-κB pathway, where Nrf2 activation can indirectly suppress NF-κB activity (39), establishing the mechanistic foundation for emodin’s synergistic anti-inflammatory and antioxidant effects.

Transforming growth factor-β (TGF-β) is recognized as the “master regulator” in the pathogenesis of organ fibrosis, particularly PF (40). Through receptor binding, it activates the downstream Smad signaling pathway, promoting phosphorylation of Smad2 and Smad3. This complex subsequently translocates to the nucleus, driving transcription of mesenchymal genes and ECM genes such as COL1A1 and FN (41, 42). Studies demonstrate that emodin significantly decreased protein expression levels of TGF-β1, Smad2, Smad3, and Smad7 in lung tissue of rats with PF, directly blocking transmission of this classical pro-fibrotic signal (28, 43, 44). Emodin reduced mRNA and protein levels of Col1 and Col3, as well as serum content of Col1 and Col3 precursors P1CP and P3NP, by inhibiting the TGF-β1/Smad and EMT axis (45). Ultimately, HYP content in lung tissue was significantly reduced, directly confirming alleviation of collagen deposition (21, 26, 46). This effect likely results from the demonstrated TGF-β suppression observed in our meta-analysis. Concurrently, emodin upregulated ADAMTS-1 expression, an ECM-degrading enzyme that facilitates breakdown of excess ECM. Conversely, emodin decreased TIMP-1 mRNA levels, relieving inhibition of ECM degradation, with this dual mechanism promoting ECM remodeling and clearance (47). This mechanism was examined in one study and, while consistent with the observed reduction in HYP content across our meta-analysis, requires replication to establish causality.

Beyond classical inhibition of Smad phosphorylation, emodin enhances anti-fibrotic effects through a non-canonical regulatory mechanism: activation of Sirt1 signaling. Sirt1 is an NAD + -dependent deacetylase whose function relates to cellular metabolism and anti-aging (48). Research indicates that emodin promotes Sirt1 expression, which subsequently deacetylates Smad3. Since acetylated Smad3 possesses higher pro-fibrotic activity, Sirt1-mediated deacetylation effectively attenuates the pro-fibrotic capacity of Smad3, providing additional protection even in the pathological environment of elevated TGF-β1 (28). This suggests that emodin’s inhibition of TGF-β1/Smad signaling operates through multiple layers and dimensions. However, this mechanism was identified in a single study within our review and represents a promising non-canonical pathway that warrants investigation in additional experimental models.

Epithelial-mesenchymal transition (EMT) represents one of the critical events initiating PF, where epithelial cells lose polarity and transform into myofibroblasts capable of migration and ECM secretion, characterized by downregulation of the epithelial marker E-cadherin and upregulation of mesenchymal markers *α*-SMA and vimentin (49). Research confirms that emodin effectively inhibits EMT progression, specifically manifested by promoting E-cadherin expression while reducing expression of α-SMA, vimentin, and FSP-1 (a marker of activated fibroblasts) (47). More detailed mechanistic investigation revealed an important molecular axis through which emodin regulates EMT: the c-MYC/miR-182-5p/ZEB2 axis. ZEB2 is a core transcriptional repressor in the EMT process that drives the mesenchymal phenotype by suppressing transcription of genes such as E-cadherin. Emodin inhibits c-MYC expression, thereby upregulating miR-182-5p. Because miR-182-5p functions as a negative regulator of ZEB2, its elevation leads to significant reduction in ZEB2 protein expression, thus relieving repression of E-cadherin and decreasing transcription of mesenchymal genes like vimentin (42). Discovery of this regulatory axis provides a precise molecular mechanism for emodin’s inhibition of EMT and suggests potential crosstalk with TGF-β1’s regulation of ZEB2. While these findings are mechanistically plausible and align with our meta-analyzed reductions in fibrosis markers, the specific molecular pathways were investigated in isolated studies and could not be quantitatively synthesized. Future research should examine these mechanisms across multiple models to enable meta-analytic confirmation.

Additionally, damage and apoptosis of alveolar epithelial cells (AECs) are considered the initial signals triggering EMT. Emodin demonstrates protective anti-apoptotic effects through regulation of Bcl-2 family proteins (50). Emodin elevated expression of the anti-apoptotic protein Bcl-2 while reducing expression of the pro-apoptotic protein Bax and the apoptosis executor caspase-3, thereby protecting alveolar epithelial cells and indirectly inhibiting EMT initiation (29).

### 4.3 Limitations

An important limitation of our analysis is that while we quantitatively synthesized key pathological and biochemical outcomes, many underlying molecular mechanisms (e.g., Sirt1, c-MYC/miR-182-5p/ZEB2 axis, ADAMTS-1/TIMP-1) were investigated in only one or few studies and could not be meta-analyzed. Therefore, our discussion of these mechanisms relies on narrative synthesis rather than pooled effect estimates. Future preclinical studies should employ standardized protocols to measure these mechanistic endpoints, enabling comprehensive meta-analytic evaluation. Additionally, the mechanistic network (Figure 10) represents a theoretical framework synthesizing individual study findings rather than pathways directly validated through our meta-analysis. Although this meta-analysis preliminarily reveals the therapeutic potential of emodin against PF, we must recognize several inherent limitations. These limitations primarily involve geographic distribution of included literature, methodological quality, and heterogeneity in statistical analysis.

First, the literature included in this study exhibits marked geographic bias, with the majority of studies originating from China. This high degree of geographic concentration may limit the external validity of results, failing to adequately represent treatment effects across different ethnicities, environments, or experimental conditions. We strongly recommend that future research should pursue broader international collaboration, incorporating literature from diverse countries and regions to enhance the generalizability of study conclusions. Additionally, some early included studies may present certain methodological and technical limitations. With the rapid advancement of molecular biology techniques, contemporary pharmacological research should adhere strictly to standardized, unified experimental protocols, particularly regarding animal model establishment, intervention dosage selection, and outcome assessment procedures. This is essential for ensuring reproducibility and reliability of experimental data. At present, we advocate active utilization of multi-omics integration technologies (such as genomics, transcriptomics, and metabolomics) alongside other advanced molecular biology approaches to conduct more comprehensive mechanistic investigations of emodin, thereby providing more robust scientific evidence for clinical treatment of PF and effectively improving the efficiency of drug clinical translation.

Second, the included literature revealed deficiencies in risk of bias and methodological quality assessment. Specifically, many studies exhibited high risk regarding blinding of outcome assessors and uncertain risk concerning allocation concealment. These biases represent major factors affecting the accuracy of systematic review results. Of particular concern is that quantitative assessment of pathology forms the foundation for evaluating emodin efficacy, yet existing literature lacks a unified gold standard for grading PF severity and pathological damage (such as the use of Ashcroft and Szapiel scoring systems). To address this shortcoming, future preclinical studies should develop more rigorous and standardized experimental protocols, unify pathological assessment criteria, and strictly adhere to randomization and blinding principles to minimize resulting research bias and error.

Finally, we must candidly acknowledge inherent statistical limitations of this systematic review and meta-analysis. According to the PICO principle, meta-analysis results for certain key outcome indicators exhibited significant heterogeneity. This heterogeneity relates closely to variations in animal model selection, intervention dosage settings, outcome assessment methods, and the number of included studies among investigators. Different experimental conditions and assessment approaches exerted substantial influence on statistical results, thereby limiting our evaluation of the accuracy and reliability of meta-analysis findings.

## 5 Conclusion

This systematic review and meta-analysis synthesized current preclinical evidence regarding the therapeutic potential of emodin in rodent models of PF, clearly demonstrating that emodin exhibits marked efficacy in ameliorating the degree of PF and reducing pulmonary pathological damage. The anti-fibrotic effects of emodin are mediated synergistically through multiple key pathways, including anti-inflammatory, antioxidant, anti-apoptotic actions, ECM clearance, and EMT inhibition. However, despite notable efficacy, the robustness and reliability of study findings warrant further consideration due to potential methodological bias risks and significant heterogeneity in outcome indicators among included studies. Therefore, future research should focus on conducting animal studies with more rigorous design and unified assessment standards to further clarify the optimal dosage and long-term safety of emodin for treating PF, thereby advancing clinical practice.

## Data Availability

The original contributions presented in the study are included in the article/Supplementary material, further inquiries can be directed to the corresponding author.

## Glossary

Ac-Smad3: Acetylated smad3
ADAMTS-1: A disintegrin and metalloproteinase with thrombospondin motifs 1
*α*-SMA: Alpha-smooth muscle actin
Bax: Bcl-2-associated X protein
Bcl-2: B-cell lymphoma 2
c-MYC: Cellular myelocytomatosis oncogene
CAT: Catalase
Col1: Collagen type I
Col3: Collagen type III
E-Cad: E-cadherin
FN: Fibronectin
FSP-1: Fibroblast-specific protein 1
GSH: Glutathione
GSH-Px: Glutathione peroxidase
HO-1: Heme oxygenase-1
miR-182-5p: Microrna-182-5p
NF-κB: Nuclear factor kappa B
Nrf2: Nuclear factor erythroid 2-related factor 2
P1CP: Carboxy-terminal propeptide of type I procollagen
P3NP: Amino-terminal propeptide of type III procollagen
p-IκBα: Phosphorylated inhibitor of kappa B alpha;
Smad2/3/7: Mothers against decapentaplegic homolog 2/3/7
Sirt1: Sirtuin 1
TIMP-1: Tissue inhibitor of metalloproteinase-1
TUNEL: Terminal deoxynucleotidyl transferase dUTP nick end labeling
ZEB2: Zinc finger E-box binding homeobox 2
