# Supplementary material for: Emodin for pulmonary fibrosis: a systematic review and meta-analysis of efficacy and molecular mechanisms
Source: Front Med (Lausanne). 2026 Jan 9;12:1734512. doi: 10.3389/fmed.2025.1734512 (PMC12828986; doi:10.3389/fmed.2025.1734512)
Supplement: Supplementary file 1 [file Data_Sheet_1.DOCX]

Supplementary Material

# Supplementary Figures

## Supplementary Figure S1


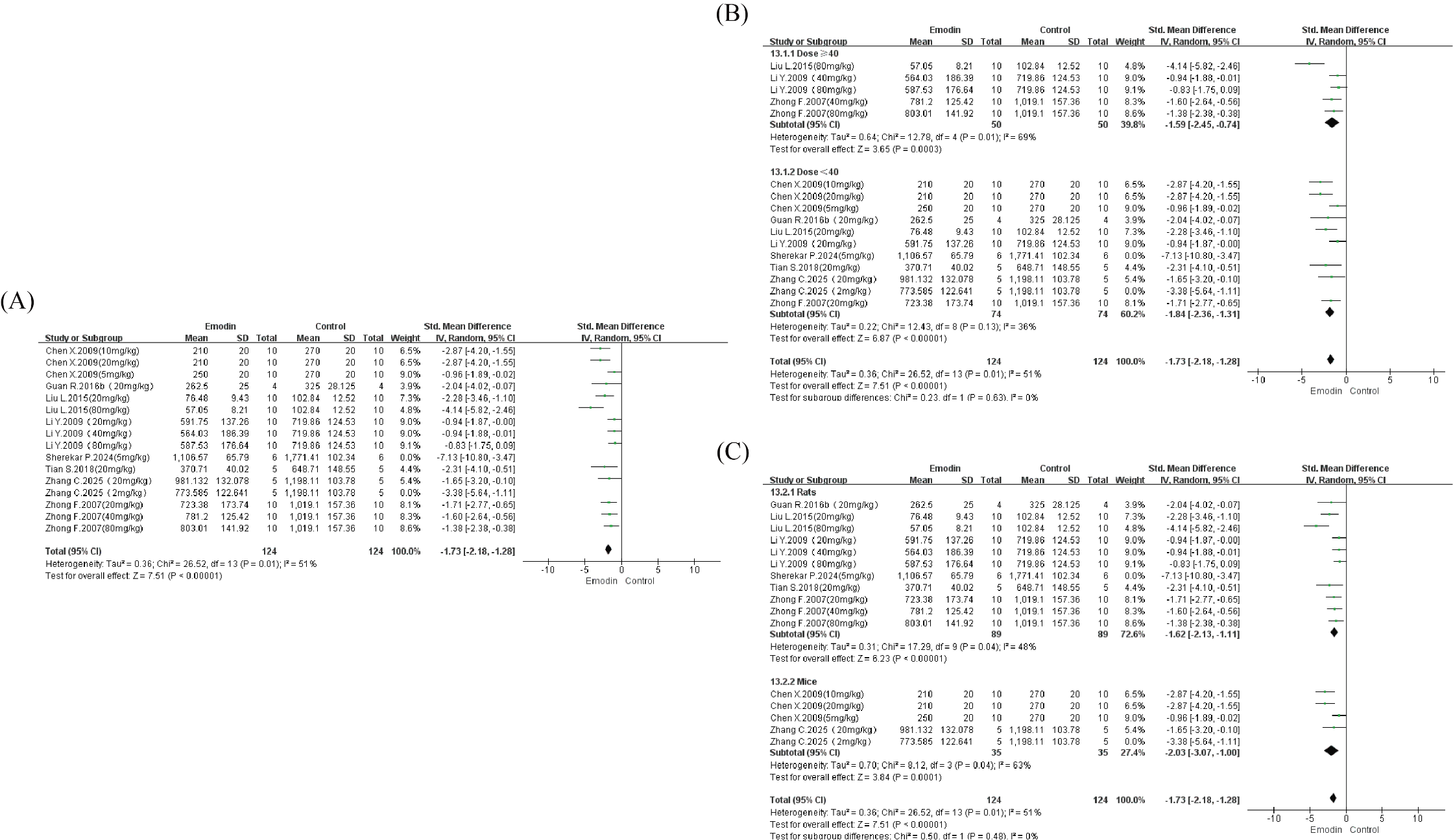


Supplementary Figure S1. Forest map for evaluating HYP content. Sensitivity analysis excluding two low-dose studies with different administration routes and formulations (Sherekar et al., 2024; Zhang et al., 2025) reduced heterogeneity across subgroups.

Abbreviations: HYP, hydroxyproline.

Note: Forest map of HYP content (A); Dose subgroup of HYP (B); Species subgroup of HYP (C).

## Supplementary Figure S2


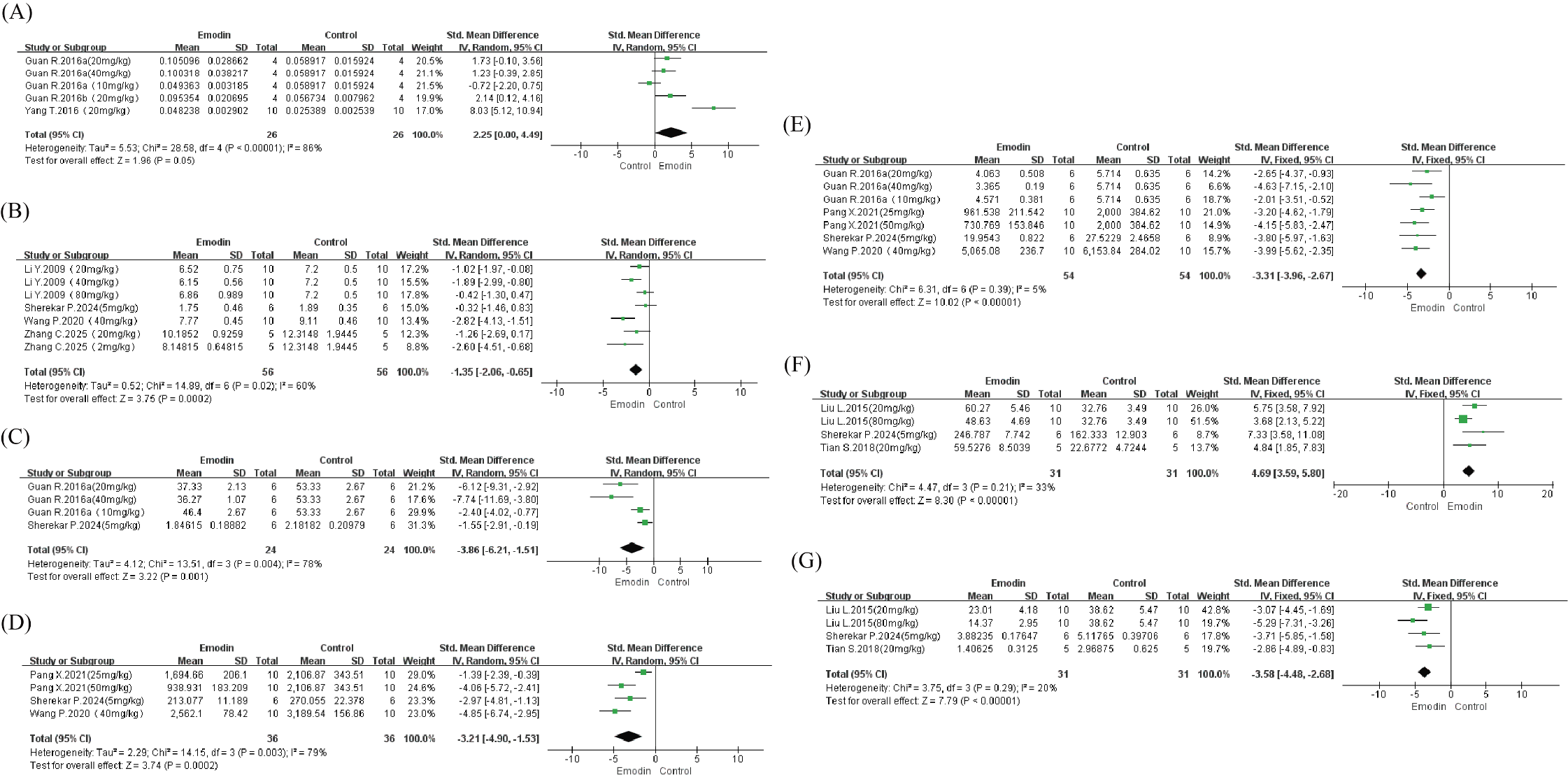


Supplementary Figure S2. Forest maps used to evaluate other outcome indicators.

Abbreviations: IL-6, interleukin-6; TNF-α, tumor necrosis factor-α; IL-1β, interleukin-1β; SOD, superoxide dismutase; MDA, malondialdehyde.

Note: Pulmonary dynamic compliance (A); Lung coefficient (B); IL-6, IL-1β and TNF-α (C-E); SOD and MDA (F, G). If the nature of the outcome indicators is not explicitly mentioned in the Supplementary Material, they are all regarded as protein content.

## Supplementary Figure S3


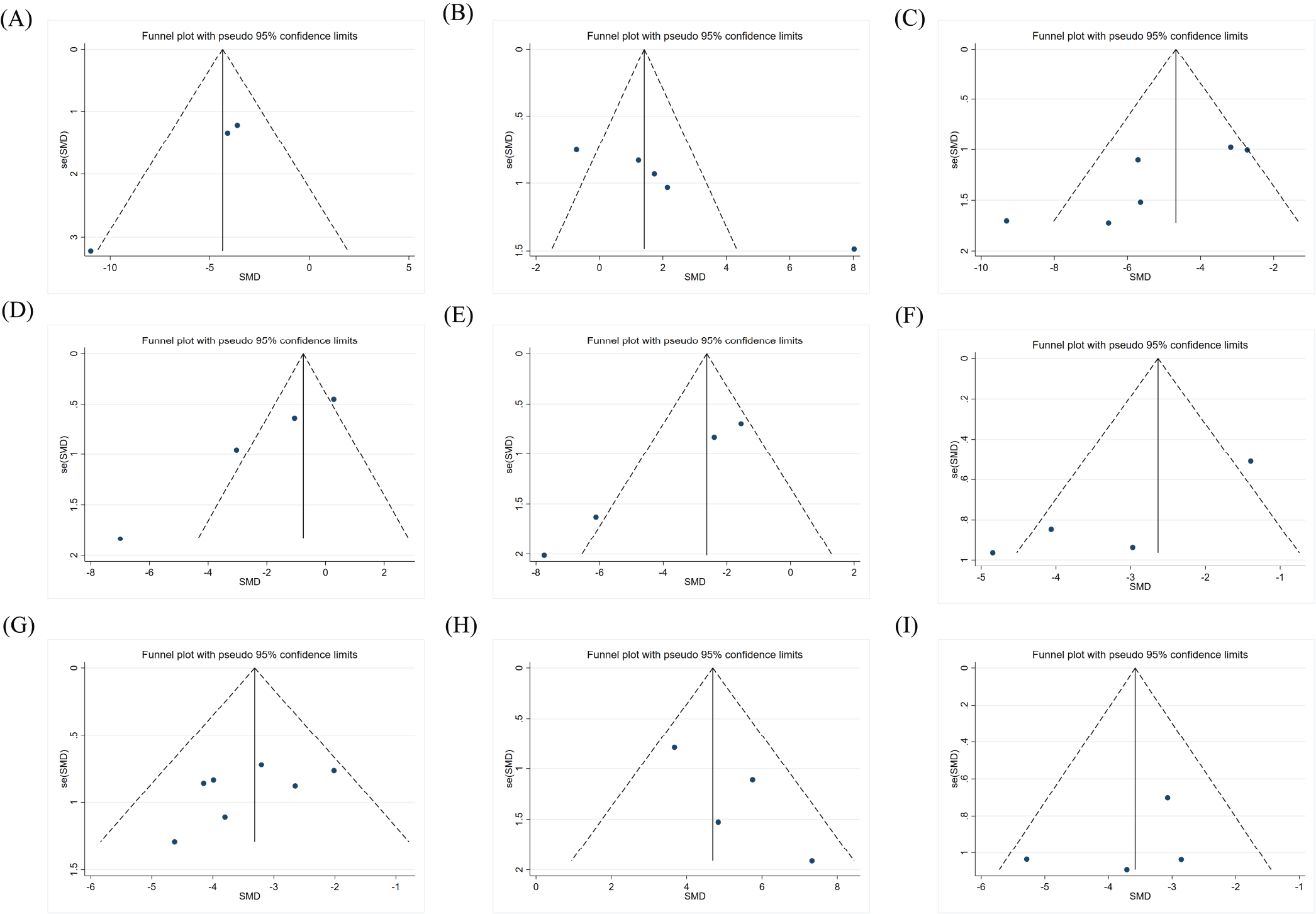


Supplementary Figure S3. Funnel plots for evaluating publication bias in other outcome measures.

Note: Percentage of fibrotic area (A); Pulmonary dynamic compliance (B); Level of TGF-β mRNA in lung tissue (C); Content of TGF-β in BALF (D); IL-6 (E); IL-1β (F); TNF-α (G); SOD (H); MDA (I).

## Supplementary Figure S4


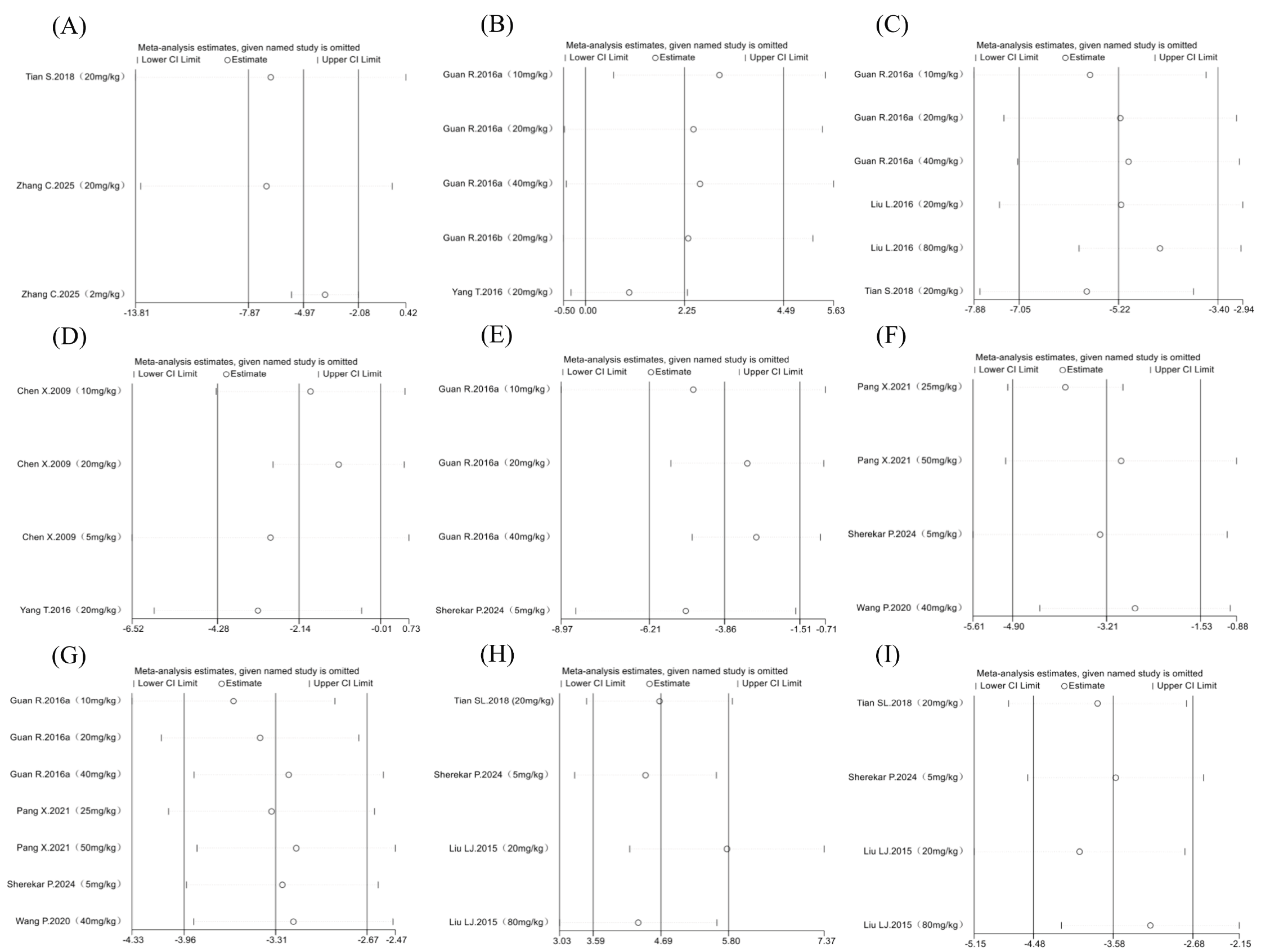


Supplementary Figure S4. Sensitivity analysis of other outcome indicators.

Note: Percentage of fibrotic area (A); Pulmonary dynamic compliance (B); Level of TGF-β mRNA in lung tissue (C); Content of TGF-β in BALF (D); IL-6 (E); IL-1β (F); TNF-α (G); SOD (H); MDA (I).
