# Supplementary material for: Emodin for pulmonary fibrosis: a systematic review and meta-analysis of efficacy and molecular mechanisms
Source: Front Med (Lausanne). 2026 Jan 9;12:1734512. doi: 10.3389/fmed.2025.1734512 (PMC12828986; doi:10.3389/fmed.2025.1734512)
Supplement: Supplementary file 2 [file Data_Sheet_2.DOCX]

Supplementary Material

# Supplementary Data

Search strategy in 8 Databases ((PubMed, Embase, Web of Science, Cochrane Library, CNKI, Wanfang, VIP, and Chinese Biomedical Database), and they are presented respectively in the form of tables.

## Search strategy in PubMed

| Steps | Search terms | Results |
| --- | --- | --- |
| #1 | "Pulmonary Fibrosis"[Mesh] | 30891 |
| #2 | ((((((((Pulmonary fibrosis[Title/Abstract]) OR (Fibroses, Pulmonary[Title/Abstract])) OR (Fibrosis, Pulmonary[Title/Abstract])) OR (Pulmonary Fibroses[Title/Abstract])) OR (Alveolitis, Fibrosing[Title/Abstract])) OR (Alveolitides, Fibrosing[Title/Abstract])) OR (Fibrosing Alveolitides[Title/Abstract])) OR (Fibrosing Alveolitis[Title/Abstract])) OR (Idiopathic Diffuse Interstitial Pulmonary Fibrosis[Title/Abstract]) | 55255 |
| #3 | Search #1 OR #2 | 63959 |
| #4 | "Emodin"[Mesh] | 1937 |
| #5 | ((((((((((Emodin[Title/Abstract]) OR (Rheum Emodin[Title/Abstract])) OR (Emodin, Rheum[Title/Abstract])) OR (Archin[Title/Abstract])) OR (Frangula Emodin[Title/Abstract])) OR (Emodin, Frangula[Title/Abstract])) OR (Peristim[Title/Abstract])) OR (1, 3, 8-trihydroxy-6-methylanthraquinone[Title/Abstract])) OR (6-methyl-1,3,8-trihydroxyanthraquinone[Title/Abstract])) OR (Emodine[Title/Abstract])) OR (Emodol[Title/Abstract]) | 3342 |
| #6 | Search #4 OR #5 | 3666 |
| #7 | Search #3 AND #6 | 19 |

## Search strategy in Web of Science

| Steps | Search terms | Results |
| --- | --- | --- |
| #1 | "Pulmonary fibrosis" (Topic) OR "Fibroses, Pulmonary" (Topic) OR "Fibrosis, Pulmonary" (Topic) OR "Pulmonary Fibroses" (Topic) OR "Alveolitis, Fibrosing" (Topic) OR "Alveolitides, Fibrosing" (Topic) OR "Fibrosing Alveolitides" (Topic) OR "Fibrosing Alveolitis" (Topic) OR "Idiopathic Diffuse Interstitial Pulmonary Fibrosis" (Topic) | 42600 |
| #2 | "Emodin" (Topic) OR "Rheum Emodin" (Topic) OR "Emodin, Rheum" (Topic) OR "Archin" (Topic) OR "Frangula Emodin" (Topic) OR "Emodin, Frangula" (Topic) | 4571 |
| #3 | #1 AND #2 | 30 |

## Search strategy in Embase

| Steps | Search terms | Results |
| --- | --- | --- |
| #1 | 'lung fibrosis'/exp OR 'lung fibrosis' | 113470 |
| #2 | 'Pulmonary fibrosis':ab,ti OR 'Fibroses, Pulmonary':ab,ti OR 'Fibrosis, Pulmonary':ab,ti OR 'Pulmonary Fibroses':ab,ti OR 'Alveolitis, Fibrosing':ab,ti OR 'Alveolitides, Fibrosing':ab,ti OR 'Fibrosing Alveolitides':ab,ti OR 'Fibrosing Alveolitis':ab,ti OR 'Idiopathic Diffuse Interstitial Pulmonary Fibrosis':ab,ti | 47856 |
| #3 | Search #1 OR #2 | 118809 |
| #4 | 'emodin'/exp OR 'emodin' | 6870 |
| #5 | 'Emodin':ab,ti OR 'Rheum Emodin':ab,ti OR 'Emodin, Rheum':ab,ti OR 'Archin':ab,ti OR 'Frangula Emodin':ab,ti OR 'Emodin, Frangula':ab,ti OR 'Peristim':ab,ti OR '1, 3, 8-trihydroxy-6-methylanthraquinone':ab,ti OR '6-methyl-1, 3, 8-trihydroxyanthraquinone':ab,ti OR 'Emodine':ab,ti OR ' Emodol':ab,ti | 4217 |
| #6 | Search #4 OR #5 | 6893 |
| #7 | Search #3 AND #6 | 55 |

## Search strategy in Cochrane Library

| Steps | Search terms | Results |
| --- | --- | --- |
| #1 | MeSH descriptor: [Pulmonary Fibrosis] explode all trees | 940 |
| #2 | (‘Pulmonary fibrosis’ OR ‘Fibroses, Pulmonary’ OR ‘Fibrosis, Pulmonary’ OR ‘Pulmonary Fibroses’ OR ‘Alveolitis, Fibrosing’ OR ‘Alveolitides, Fibrosing’ OR ‘Fibrosing Alveolitides’ OR ‘Fibrosing Alveolitis’ OR ‘Idiopathic Diffuse Interstitial Pulmonary Fibrosis’):ti,ab,kw | 4819 |
| #3 | Search #1 OR #2 | 4830 |
| #4 | MeSH descriptor: [Emodin] explode all trees | 4 |
| #5 | (Emodin):ti,ab,kw OR (Rheum Emodin):ti,ab,kw OR (Emodin, Rheum):ti,ab,kw OR (Archin):ti,ab,kw OR (Frangula Emodin):ti,ab,kw (Word variations have been searched) | 15 |
| #6 | (Peristim):ti,ab,kw OR (Emodin, Frangula):ti,ab,kw OR (Emodine):ti,ab,kw OR (Emodol):ti,ab,kw (Word variations have been searched) | 17 |
| #7 | Search #4 OR #5 OR #6 | 17 |
| #8 | Search #3 AND #7 | 0 |

## Search strategy in CNKI

| Steps | Search terms | Results |
| --- | --- | --- |
| #1 | ((SU%=肺纤维化 OR SU%=特发性肺纤维化 OR SU%=石棉肺) OR (TKA=肺纤维化 OR TKA=特发性肺纤维化 OR TKA=石棉肺)) AND ((SU%=大黄素 OR SU%=朱砂莲甲素 OR SU%=1, 3, 8-三羟基-6-甲基蒽醌 OR SU%=泻素) OR (TKA=大黄素 OR TKA=朱砂莲甲素 OR TKA=1, 3, 8-三羟基-6-甲基蒽醌 OR TKA=泻素)) | 37 |

## Search strategy in Wanfang Database

| Steps | Search terms | Results |
| --- | --- | --- |
| #1 | (主题:(肺纤维化) or 主题:(特发性肺纤维化) or 主题:(石棉肺)) and (主题:(大黄素) or 主题:(朱砂莲甲素) or 主题:(1, 3, 8-三羟基-6-甲基蒽醌) or 主题:(泻素)) | 114 |

## Search strategy in VIP Database

| Steps | Search terms | Results |
| --- | --- | --- |
| #1 | ((M=肺纤维化 OR M=特发性肺纤维化 OR M=石棉肺) OR (R=肺纤维化 OR R=特发性肺纤维化 OR R=石棉肺)) AND ((M=大黄素 OR M=朱砂莲甲素 OR M=1, 3, 8-三羟基-6-甲基蒽醌 OR M=泻素) OR (R=大黄素 OR R=朱砂莲甲素 OR R=1, 3, 8-三羟基-6-甲基蒽醌 OR R=泻素)) | 112 |

## Search strategy in China Biology Medicine (CBM) Database

| Steps | Search terms | Results |
| --- | --- | --- |
| #1 | "肺纤维化"[不加权:扩展] | 15475 |
| #2 | "肺纤维化"[常用字段:智能] OR "特发性肺纤维化"[常用字段:智能] OR "石棉肺"[常用字段:智能] | 58895 |
| #3 | Search #1 OR #2 | 58895 |
| #4 | "大黄素"[不加权:扩展] | 3972 |
| #5 | "大黄素"[常用字段:智能] OR "朱砂莲甲素"[常用字段:智能] OR "1,3,8-三羟基-6-甲基蒽醌"[常用字段:智能] OR "泻素"[常用字段:智能] | 9740 |
| #6 | Search # 4 OR # 5 | 9740 |
| #7 | Search # 3 AND # 6 | 28 |
