# Supplementary material for: Emodin for pulmonary fibrosis: a systematic review and meta-analysis of efficacy and molecular mechanisms
Source: Front Med (Lausanne). 2026 Jan 9;12:1734512. doi: 10.3389/fmed.2025.1734512 (PMC12828986; doi:10.3389/fmed.2025.1734512)
Supplement: Supplementary file 3 [file Table_1.DOCX]

Supplementary Material

**Supplementary Table**

Egger test

| **Outcome** | **t** | **p** | **95% Conf. interval** | | **No. of studies (containing different doses)** |
| --- | --- | --- | --- | --- | --- |
| Percentage of fibrotic area | -94.96 | 0.007 | -4.16793 | -3.184215 | 3 |
| Level of TGF-β mRNA in lung tissue | -3.11 | 0.036 | -11.25432 | -0.6378148 | 6 |
| Content of TGF-β in BALF | -10.24 | 0.009 | -8.01981 | -3.275642 | 4 |
| Pulmonary dynamic compliance | 7.62 | 0.005 | 6.486226 | 15.79899 | 5 |
| IL-6 | -25.80 | 0.001 | -5.528836 | -3.948379 | 4 |
| IL-1β | -3.27 | 0.082 | -14.42478 | 1.973947 | 4 |
| TNF-α | -1.45 | 0.206 | -8.174603 | 2.266426 | 7 |
| SOD | 2.11 | 0.169 | -2.833014 | 8.301656 | 4 |
| MDA | -0.76 | 0.527 | -16.1208 | 11.28158 | 4 |

Abbreviations: TGF-β, transforming growth factor-β; IL-6, interleukin-6; TNF-α, tumor necrosis factor-α; IL-1β, interleukin-1β; SOD, superoxide dismutase; MDA, malondialdehyde.
